# Harnessing the power of resistant starch: a narrative review of its health impact and processing challenges

**DOI:** 10.3389/fnut.2024.1369950

**Published:** 2024-03-20

**Authors:** Nathália Trunckle Baptista, Robin Dessalles, Anne-Kathrin Illner, Patrice Ville, Léa Ribet, Pauline M. Anton, Mickaël Durand-Dubief

**Affiliations:** ^1^Baking Science and Nutrition, Lesaffre Institute of Science and Technology, Lille, France; ^2^Robin Dessalles EI, Lyon, France; ^3^Transformations and Agroressources, Institut Polytechnique UniLaSalle, Université d’Artois, Beauvais, France; ^4^Department of Regulatory Department, University of Lesaffre International, Marcq-en-Baroeul, France; ^5^Discovery and Front-End Innovation, Lesaffre Institute of Science and Technology, Lille, France

**Keywords:** resistant starch, human health, food processing, nutritional properties, fibers

## Abstract

Starch is a primary energy storage for plants, making it an essential component of many plant-based foods consumed today. Resistant starch (RS) refers to those starch fractions that escape digestion in the small intestine and reach the colon where they are fermented by the microflora. RS has been repeatedly reported as having benefits on health, but ensuring that its content remains in food processing may be challenging. The present work focuses on the impact RS on health and explores the different processes that may influence its presence in foods, thus potentially interfering with these effects. Clinical evidence published from 2010 to 2023 and studying the effect of RS on health parameters in adult populations, were identified, using PUBMED/Medline and Cochrane databases. The search focused as well on observational studies related to the effect of food processes on RS content. While processes such as milling, fermentation, cooking and heating seem to have a deleterious influence on RS content, other processes, such as cooling, cooking time, storage time, or water content, may positively impact its presence. Regarding the influence on health parameters, there is a body of evidence suggesting an overall significant beneficial effect of RS, especially type 1 and 2, on several health parameters such as glycemic response, insulin resistance index, bowel function or inflammatory markers. Effects are more substantiated in individuals suffering from metabolic diseases. The effects of RS may however be exerted differently depending on the type. A better understanding of the influence of food processes on RS can guide the development of dietary intake recommendations and contribute to the development of food products rich in RS.

## Graphical abstract

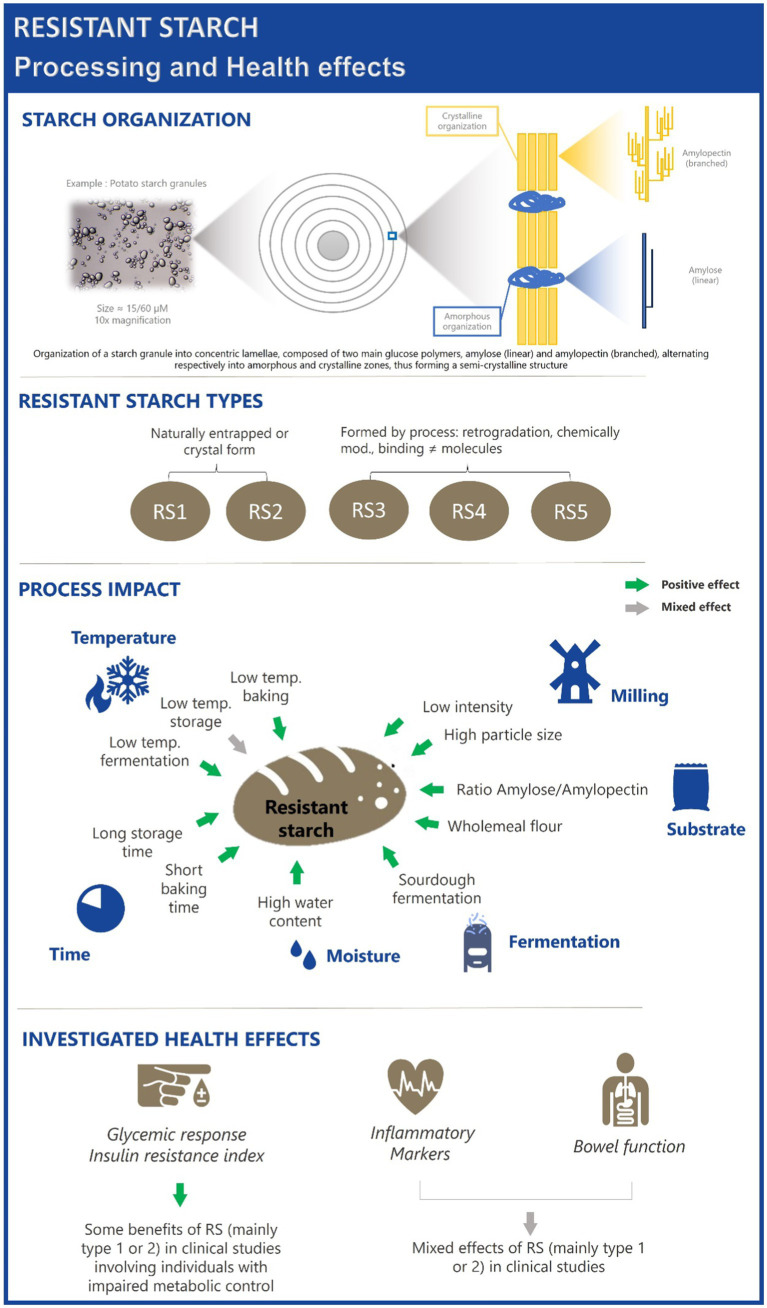

## Introduction

Carbohydrates (CHO) are fundamental components of human nutrition, serving as the primary source of energy in most diets. Additionally, they play a significant role in human health through their influence on glucose homeostasis via their metabolism in the gastrointestinal tract. Carbohydrates are abundantly found in cereals, fruits, vegetables and legumes. Among them, starch is the most abundant digestible polysaccharide in human diets, playing a crucial role in numerous plant-based foods such as wheat, maize, rice, rye, potato or peas (1, 2). Starches are classified based on their digestion rates into three major categories: rapidly digestible starch (RDS), slowly digestible starch (SDS) and resistant starch (RS). RDS and SDS are both fully digested in the small intestine, though at different rates: within 20 min for the former versus 120 min for the latter (3–5). In contrast, RS mostly escapes digestion in the small intestine and is fermented in the colon by microorganisms, producing short-chain fatty acids (SCFA) (3, 4, 6). This metabolic pathway looks like that of dietary fibers, plant components, originally defined as “*portion of food which is derived from cellular walls of plants which are digested very poorly by human beings*.” This led to an expansion of the definition to encompass RS along with other compounds such as resistant oligosaccharides or hydrocolloids (7). Dietary fibers have long been recognized as an important component of a healthy diet and are emphasized in dietary guidelines worldwide. For example, a daily intake of at least 25 grams per day is recommended in Europe to reduce the risk of chronic diseases (1). However, actual intake of dietary fibers in Europe varies significantly across countries, and often falls short of these recommendations. Reports indicate an average intake of 12.5 g/d in Spain, 17 g/d per day in Belgium, 20 g/d in France, Sweden, and the Netherlands, 21 g/d in Austria and Finland, with only Norway and Germany approaching the recommended 25 g/d (8). In the United States, it was estimated that only 7.4% of adults reached the recommended intake of 14 g/1000 kcal (9). In Australia, a typical diet provides 4 times less than the 15–20 g/day recommended for supporting bowel health (10). More limited data are available regarding RS consumption. Global intake is estimated to be between 3 and 10 g per day. In Europe, intake ranges from approximately 3 to 6 g/d, while in China, it is about 15 g/d, mainly from wheat and rice products. In Africa, intake can be as high as 20–30 g/d (11).

Apart from its contribution to fiber intake, increasing the RS content in foods may be an opportunity to promote health since starch microstructure manipulation may modulate health parameters such as those related to metabolic response. However, the influence of food processing on RS content appears as a key determinant in this respect.

In the present work, we aim to describe the influence of various food processes on RS content, and to review clinical evidence related to the effects of RS on health, in order to highlight the barriers and knowledge gaps that remains to be addressed before developing public health strategies.

## Methods

To compile this narrative review, a comprehensive literature search was conducted on 3 scientific databases: Medline, COCHRANE, and The Lens. The focus was on articles examining the health benefits of RS and the dietary processes that may influence its content in foods were searched. The keyword “resistant starch” was combined with others, related to health conditions, as follows: “resistant starch” AND (“glycemic response” OR “glycaemic response” OR diabetes OR diabetics OR metabolic OR overweight OR obese OR glucose OR insulin OR lipids OR inflammation OR microbiota OR microflora OR gastrointestinal OR oxidative OR antioxidant). Searches with keywords related to food processes were performed in parallel: “resistant starch” AND (cooking OR cooling OR milling OR storage OR heating OR breeding OR microwave). The retrieved records were screened by reviewers, and studies were selected based on title, abstract and keywords, according to the following eligibility criteria: peer-review publications in English, published in the last 20 years, either randomized controlled trials, systematic reviews, meta-analyses or observational trials, conducted in the general adult population. Studies conducted on tertiary-care patients, such as those suffering from cancer or undergoing hemodialysis were excluded. Cross-reference searches were performed during the selection and reviewing processes. Following selection by the authors, full-text articles were retrieved and analyzed.

### Types of resistant starch and their properties

RS is usually listed in five categories (see Table 1). Resistant starch type 1 (RS1) is physically entrapped, non-accessible, in a non-digestible matrix such as whole grains (intact cells) due to wall barrier. This starch is heat-stable and does not break during cooking, but it does during milling. It is commonly found in products such as whole bread, seeds or legumes. Resistant starch type 2 (RS2), is a native granular RS found in raw potatoes or green bananas. Its crystalline organization protects it from digestive enzymes. RS2 has been widely investigated in clinical trials, notably through the use of HI-MAIZE® 260 (Ingredion United Kingdom, Ltd) as an additive to the test products. Resistant starch type 3 (RS3) is also known “retrograded starch” which results from the formation of double helixes by long-branch chains of amylopectin following the cooling of foods cooked in the presence of moisture. It cannot be hydrolysed by digestive enzymes. Resistant starch type 4 (RS4) is a chemically-modified starch engineered to resist to enzymatic digestion. As well as RS2, it can be added to foods as an ingredient. Products containing RS4 might be derived from sources such as potato, high-amylose maize, or tapioca starches (VERSAFIBE™ 1,490, VERSAFIBE™ 2,470 or NOVELOSE®3,490, respectively) (6). Initially, a fifth class of resistant starch (RS5) was established to characterize cases where RS is modified and form starch-lipid complexes with resistant properties. However, recent evidence has described the generation of starch complexes that involve other molecules, such as amino acids, peptides, polysaccharides or polyphenols, and have a similar structure to starch-lipid complexes. Therefore, an update of the “RS5” category has been suggested to include these new complexes as well (14, 15).

**Table 1:** Characteristics of resistant starch fractions (5, 12, 13).

Type of RS	Description	Food sources	Digestion in small intestine
RS 1	Non-accessible, physically entrapped	Whole or partly milled grains, seeds or legumes	Slow rate; partial degree; totally digested if properly milled
RS 2	Ungelatinized resistant granules	Raw potatoes, green bananas, some legumes, high-amylose corn, specific ingredients (e.g., HI-MAIZE® 260)	Very slow rate; little degree; totally digested when freshly cooked
RS 3	Retrograded starch	Cooked and cooled potatoes, bread, cornflakes, food products with repeated moist heat treatment	Slow rate; partial degree; reversible digestion; digestibility improved by reheating
RS 4	Chemically modified starches due to cross-linking with chemical reagents	Foods in which modified starches have been used (e.g., breads, cakes). Example of ingredients: VERSAFIBE™ 1,490, VERSAFIBE™ 2,470, NOVELOSE®3,490	Result of chemical modification; can resist hydrolysis
RS 5	Amylose-lipid complexes	Foods with high amylose content	Can resist enzymatic digestion

### Effects of food processing on resistant starch content

The preservation of starch’s crystalline structure is crucial for maintaining low digestibility to maximize their nutritional properties (low glycaemia index, satietogenic properties ….). Some foods, such as unriped bananas and uncooked oats, are naturally rich in RS. However, various factors may alter this content in one direction or another (16). These factors may be encountered at an early stage (e.g., arising from varietal selection), or later as a result of the physical treatments occurring in various steps of food processing, from milling to food storage (17).

#### Varietal differences and breeding techniques

Developing crops with a modified amylose-to-amylopectin ratio can enhance RS levels in foods. Higher amylose content promotes the retrogradation after cooking, thus regenerating RS. This is because amylose has a smaller and more flexible structure due to its linearity, making it easier to regenerate compared to amylopectin (17–20). Several studies have demonstrated that using a wheat cultivar with elevated amylose content significantly increases the final RS content in bread, compared to a conventional cultivar (18, 21–23). This elevated content seems to translate into health benefits, as the production of high-amylose rice (24), noodles (25), or wheat (26) positively impacted postprandial glycemia in several randomized controlled trials. However, it is worth noting that, using specific high-amylose cultivars may negatively impact the characteristics of dough and bread (27).

#### Milling

Milling disrupts the crystalline structure of starch, increasing its exposure to enzymatic degradation and leading to a significant loss of RS. Compared to whole grains, cereal flours, especially wheat, are typically lower in RS (12, 28, 29). Coarse milling or selecting larger particles after fractionation could attenuate this loss (19), though other production steps also significantly affect RS content (18). Additionally, whole grain products are more likely to contain RS1, as starch is encapsulated within a plant structure (28).

#### Cooking/heating

Cooking has a major influence on both starch organization and its level in foods. At or above a defined temperature called the “gelatinization temperature” (GT), and in the presence of sufficient water, the crystalline structure of the starch granule is disrupted, making starch resistant (both RS1 and RS2) more digestible. However, during cooling, starch polymers (mostly amylose), tend to reassociate, forming packed structures which remain unavailable for enzymatic hydrolysis. This process is called retrogradation (29, 30).

This new fraction, retrograded starch (RS3), is the main form of RS in processed foods (22) and the major contributor to RS intake (13).

Microwave cooking, through dielectric heating and electromagnetic effects, generally leads to retrogradation (31). Microwave reheating of rice, regardless of water content, increase RS while reducing digestible starch fractions (32, 33). This effect seems to extend to other foods such as potatoes (34). Cooking wheat noodles in a microwave was found more effective than boiling or steaming in preserving RS and lowering glycemic index (35). In a meta-analysis of 31 articles investigating the effect of microwave treatment on starch content of high-carbohydrate foods, Isra et al. highlighted that this method of cooking significantly increased the level of RS, whatever the food matrix [2.755% (95% CI: 2.106 to 3.403); *p* < 0.001]. Moreover, RS enrichment resulted in enhanced prebiotic properties, based on several parameters, namely starch composition, amylose interaction, lactic acid bacteria viability, and enteropathogenic *Escherichia coli* viability (36).

Control over starch-to-moisture ratio, temperature, and heating time can significantly alter the resulting starch levels (37). For example, heat-moisture treatment (HMT) and annealing are two hydrothermal treatments commonly used to alter starch properties (38). HMT consists in heating the starch granule at high temperature (ranging from 84 to 140°C) while maintaining low moisture (10 to 35%) for specific period of time, in order to prevent gelatinization. Studies assessing the effects of different HMT treatments on starch characteristics have been extensively reviewed (38). Recently, progressive increase in temperature during HMT has been shown to reduce the digestibility of sweet potato starch by decreasing RDS and increasing RS fractions, with optimum conditions at 110°C, 25% moisture for 4 h (39). Similar results were observed in rice starch (40, 41), and starch from other foods under various conditions (38). Subjecting barley to HMT has been reported to improve glycemic response and to promote the growth of SCFA-producing bacteria in rats (42). Overall, the ability to modify starch swelling capacity, crystallinity, gelatinization or retrogradation, digestibility through HMT gives an indication of its potential to impact metabolic response to foods and thus, its benefits on health.

Modifications of RS crystallinity and organization may also be obtained through the treatment of starch with excess water and limited temperature, using a process called annealing. Typical treatment conditions are around 50°C temperature for 24 h or more (38). However, the ability of annealing to modify the characteristics of starch seems dependent on the botanical source: Zheng et al. recently evidenced increased crystallinity of maize and potato starch with increasing time of annealing treatment, while the effect on pea starch was limited. An increase in RS together with a decrease in RDS were noted after potato starch treatment, while no such changes were observed in the case of maize or pea starch (43). In another study, a decreased in proso millet starch digestibility was observed after annealing without modifications of the crystallinity (44). Annealing was found to raise RS content of cornstarch by almost 9-fold, and was shown to be more effective in this respect than HMT, autoclaving or microwave treatments (45). Therefore, the use of annealing to enhance starch functional properties needs cautious selection of the appropriate conditions and substrate.

Autoclaving (i.e., high temperature, high-pressure treatment) is also known to induce RS3 formation, especially when coupled to cooling (4). Based on a meta-analysis of 10 studies, Faridah et al. highlighted that the effect of autoclaving-cooling treatment was dependent on the food source, the water ratio, as well as treatment time and temperature. Thus, using corn, oat or rice as food source, having a starch-to-water ratio of 1:4 and performing two cycles of autoclaving-cooling for 30 min of autoclaving at 121°C were the conditions allowing maximization of the RS content (46).

Long and low-temperature bread baking (e.g., pumpernickel conditions: 20 h, 120°C), significantly increases RS content compared to standard baking (45 min at 200°C) (18, 21–23). For instance, white wheat bread baked at 120°C for 4 h, and another at 150°C for 3 h, showed, respectively, a 24% (1.46 g/100 g) and 15% (1.36 g/100 g) increase in RS content, compared to the same bread baked for 30 min at 200°C (1.18 g/100 g) (30).

#### Cooling/storage

Storage conditions, particularly cooling after cooking, play key role for the RS content in foods. Cooling after cooking is a crucial step during which starch retrogrades, partially restoring its crystallinity (17, 28). In a study, freshly baked bread was stored at ambient (20°C), frozen (− 17°C) or refrigerated (3.5°C) temperatures for 7 days; authors reported that the RS content was significantly increased in the refrigerated bread compared to the other storage conditions (47). Cooling white rice for 24 h at 4°C before reheating resulted in a higher RS content of 1.65 g/100 g compared to the rice cooled at ambient temperature for 10 h. In addition, the glycemic response was significantly reduced compared to freshly cooked white rice in healthy individuals (48). Similar preparation conditions also showed a significant reduction of postprandial glycemia in type 1 diabetics compared to rice served immediately after cooking (49). Another study noted that the impact of refrigeration on RS content differs depending on the rice variety. Maximization of RS content was attained when long-grain rice was prepared using a rice cooker and then refrigerated for 3 days at 4°C (2.55 g RS per 100 g). In contrast, short-grain rice prepared in a pressure cooker and similarly refrigerated showed the lowest RS dose among the tested combinations (0.20 g RS/100 g rice) (50). The influence of storage time and temperature on RS content appears to be food-specific: RS content in noodles was maximized when microwave-heated and stored for 48 h at room temperature (51).

One study highlighted a steady increase in RS content in sourdough teff breads over 5 days of storage, with retrogradation was evidenced by a concomitant decrease of RDS (52). The effect of storage time on RS content, regardless of temperature, has been observed in wheat bread as well (30). Interestingly, the rate of starch recrystallization during storage varies among cereals. For instance, rye sourdough bread exhibits a slower crystallization rate than wheat bread (53).

#### Fermentation conditions

Several studies reported an effect of fermentation conditions on the RS content of foods.

One study found that sourdough bread, regardless of flour type (whole or white wheat), had a significantly higher RS content compared to yeast bread. Furthermore, at the same fermentation temperature, using type-2 sourdough fermentation using indigenous strains (*Lactobacillus brevis* ELB99, *Lactiplantibacillus plantarum* ELB75, and *Saccharomyces cerevisiae* TGM55) yielded higher RS content in bread compared to type-1 (spontaneous) fermentation. In white wheat bread, lower fermentation temperature (25°C vs. 30°C) also increased the RS content of bread, whatever the fermentation type. In whole wheat bread, this was the case only for type-1 fermentation (54). In contrast, another study did not find any significant impact of sourdough addition on the RS in white flour (30). In the case of Teff bread, Shumoy et al. reported that the RS content increased with the proportion of incorporated sourdough (52).

In experiments involving barley malt production, Teixeira et al. highlighted that the increase in RS was correlated to the barley variety used, particularly its amylose content. Specifically, steeping with 0.4% lactic acid in Tipple, regardless of the temperature used, was shown to enhance RS content (55).

### Impact of resistant starch on health based on a clinical perspective

The effects of RS on health have been widely investigated in the past decades. For this review, a total of 14 meta-analyses compiling data from these studies were retrieved (56–70). These meta-analyses vary in their focus on different types of RS and target populations. The following sections will review and summarize the impact of RS on different health parameters. Characteristics of the meta-analyses are detailed in Table 2.

**Table 2:** Characteristics of included meta-analyses.

Studies characteristics												Results
Reference	Nb of studies	Type	Design	Total nb of subjects	Target population	Intervention	Control	RS dose range (g/d)	Study duration range (weeks)	RS type	Outcomes	
(59)	14	RCT	// or CO	515	T2DM or obesity w/o T2DM	RS supplementation	non-RS supplementation	4–6	4–52	Any	fasting insulin, fasting glucose, BMI and HOMA-IR;	↓ FPG in T2DM + OB; ↓ FPI in T2DM + OB; = FPI in T2DM; = BMI in T2DM; ↓ HOMA-IR in T2DM + OB; = HOMA-IR in T2DM
(61)	19	RCT	// or CO	1,014	Any	RS supp or intervention	intake of digestible starches or other CHOs	8–34	3–48	Any (mostly RS2)	glycemic status, serum lipoproteins and inflammatory markers	↓ FPG; ↓ FPI; = HOMA-IR; ↓ HbA1c; ↓TC; ↓ LDL; = HDL; = TAG; = IL-6; ↓ TNF-α; = CRP
(64)	31	RCT	Any	982	T2DM or prediabetes; no other chronic conditions	Resistant starch type 1–5, starch-containing foods	Starch ot other carbohydrates	1–45	0–52	Any	Markers of glycemia	↓ PPG; = PPI; ↓ FPG; ↓ FPI
(66)	22	RCT	Any	670	Healthy or MetS or T2DM	Resistant starch type 2	Any	8–66	1–12	RS2 (mostly high amylose maize starch)	fasting blood glucose, glycated hemoglobin (HbA1c), insulin resistance, appetite/satiety levels, lipid levels or body weight	= FPG, ↓ body weight; = HOMA-IR; = HbA1c; = TC; = LDL; = HDL; = TAG
(70)	15	RCT	Any	503	Any	resistant starch supplementation (excluding type 1)	Any	5,1–66	2–12	Any (mostly RS2)	blood glucose or insulin	↓ FPG; = FPI; = HbA1c;
(68)	13	RCT	Any	428	Overweight / Obesity	RS	Any	10–45	2–12	Any	fasting glucose or fasting insulin or plasma lipid or insulin sensitivity or insulin resistance	↓ FPG; ↓ FPI; = HOMA-IR; ↓ HbA1c; = TC; ↓ LDL; = HDL; = TAG
(71)	14	RCT	Any	820	Adults, healthy or not,no lipid-lowering medication	RS	placebo without the functions of DF or diets low in resistant starch	10–66	2–52	Any (mostly RS2)	TC, LDL-C, TGs, HDL	↓ TC; ↓ LDL; = HDL; =TAG
(60)	8	RCT	Any	308	Any but inflammatory diseases	RS2	Placebo	10–45	4–12	RS2 (Hi-maize 260)	IL-6, hs-CRP, TNF-a as outcomes	= IL-6; = TNF-α; = CRP
(63)	16	RCT	Any	706	Any	RS alone	Any	6–27	4–12	RS2	Inflammatory or oxidative stress biomarkers	↓ IL-6 in non-T2DM; ↓ TNF-α in non-T2DM; ↓ CRP in T2DM; = uric acid; ↑ TAC; ↑ SOD; ↓ MDA
(67)	13	RCT	Any	672	Adults, healthy or not	Resistant Starch (“resistant maltodextrin,” “resistant dextrin,” “indigestible starch,” “high amylose starch”)	Any but RS	10–45	4–14	Any (mostly RS2)	IL-6, CRP, hs-CRP and TNF-α	↓ IL-6; ↓ TNF-α; = CRP
(69)	16	RCT	Any	739	Adults	RS	Any	Any	2–12	Any	IL-6, CRP, hs-CRP and TNF-α	↓ IL-6; ↓ TNF-α; = CRP; ↑ TAC; = SOD; = MDA
(65)	9	RCT	Any	193	Healthy adults	RS	Any	22–45 (avg 33)	1–4	RS2, RS3 and RS4	butyrate concentration, defecate frequency, fecal wet weight, fecal PH	↑ stool volume; = stool frequency; ↑ butyrate levels; ↓ fecal pH
(57)	7	Any	Any	248	Any	RS	Baseline	Any	NA	Any	microbial diversity, bacterial counts	↓ gut microbial α-diversity; ↑ counts of *Ruminococcus, Agathobacter, Faecalibacterium and Bifidobacterium*
(72)	29	RCT	SB or DB	1,208	Healthy adults	RMD	Any w/o RMD	3,8-13,5	1–3	RMD	stool frequency and stool volume	↑ stool volume; ↑ stool frequency
(73)	20	RCT	Any		Colorectal cancer, familial adenomatous polyposis (FAP), Lynch syndrome (LS), sporadic adenoma (SA) and healthy subjects	RS/inulin with or without other drugs	Digestible carbohydrate or low-dose NDC	10–60	4–116	Any	SCFA production	= SCFAs; = butyrate

BMI, body mass index; CRP, c-reactive protein; FPG, fasting plasma glucose; FPI, fasting plasma insulin; GIP, glucose-dependent insulinotropic polypeptide; GLP-1, glucagon-like peptide 1; HbA1c, glycated hemoglobin; HDL, high-density lipoproteins cholesterol; HOMA-IR, homeostatic model assessment of insulin resistance; IL-6, interleukin 6; LDL, low-density lipoprotein cholesterol; MDA, malondialdehyde; PPG, postprandial glucose; PPI, postprandial insulin; SOD, superoxide dismutase; TAC, total antioxidant capacity; TAG, triacylglycerol; TC, total cholesterol; TNF-α, tumor necrosis factor alpha; ↓: significantly decreased vs. control; ↑ significantly increased vs control.

#### Resistant starch and gastrointestinal outcomes (stools characteristics, microbiota and short-chain fatty acids)

RS has been reported to impact gastrointestinal outcomes, from microbiota population counts and activity, to stools characteristics.

A meta-analysis included 9 randomized controlled trials administering RS at doses varying from 22 g/day to 45 g/day (average 33 g/day) to healthy subjects. Control groups typically followed low-RS diets. The pooled analysis revealed significant improvements in fecal weight, butyrate levels, and fecal pH following RS supplementation, compared to the control. Conversely, defecation frequency was not significantly changed (65).

In another meta-analysis (72) a significant increase in stool volume and frequency compared to the control was found based on a pooled analysis of 29 randomized controlled trials administering resistant maltodextrin (RMD) from 3.8 to 13.5 g daily to healthy individuals.

Regarding studies focusing on bacterial counts in human gut microbiota, pooled data from 7 studies involving 248 individuals revealed an association between RS consumption and an increased abundance of *Ruminococcus*, *Agathobacter*, *Faecalibacterium* and *Bifidobacterium*. RS appeared to impact mechanisms related to carbohydrate and lipid metabolism. Notably, different RS types were found to alter differently microbiome responses (57).

Focusing on gut microbiota activity, one meta-analysis reported no significant changes in total SCFAs or butyrate concentration following RS intervention, based on data from 4 and 3 studies, respectively. Similarly, total SCFAs and butyrate excretion were unchanged in the intervention group (RS2 or inulin) compared to control (placebo or digestible CHO), based on data from 5 and 3 studies, respectively. The same study also assessed the potential of non-digestible fibers in reducing the risk of colorectal cancer, but the evidence reviewed did not support this hypothesis (73).

#### Resistant starch and metabolic response (glucose, insulin)

RS may dampen glycemic and insulin responses by delaying the absorption of food boluses. This topic has been the subject of extensive research in recent years, as evidenced by 7 meta-analyses published between 2018 and 2023.

Most recently, RS was found to significantly reduce postprandial glucose following both acute [−0.65 (95% CI: −0.98, −0.32); *p* < 0.0001; 14 studies] and chronic [−0.31 (95% CI: −0.50, −0.13), *p* = 0.001; 7 studies] intake. Chronic intake of RS also led to a significant reduction in fasting blood glucose [−0.31 (95% CI: −0.51, −0.11); *p* = 0.002; 14 studies], with doses used in studies ranging from 6 to 40 g/d. Interestingly, both RS1 and RS2, but not RS3 achieved this reduction. For fasting blood glucose, significant results were observed only with chronic intake of RS2. Focusing on the target population, the authors noted that the acute postprandial glycemic response was significantly reduced in both prediabetics and type-2 diabetics, but the chronic response was effective only in diabetics subjects. Other metabolic markers such as glucagon-like peptide-1 (GLP-1), glucose-dependent insulinotropic polypeptide (GIP), and insulin sensitivity (HOMA-IR) remained unaffected by RS (64).

In another paper (70), results from a pooled analysis showed significant improvements in fasting plasma glucose (FPG) [−0.09 (95% CI –0.13, −0.04) mmol/l; *p* = 0.001; 16 trials] and insulin resistance (HOMA-IR) [−0.33 (95% CI –0.51, −0.14); *p* = 0.001; 3 trials], in 503 individuals, healthy or not, receiving RS (except type 1) compared to unsupplemented control food. However, no significant effects of RS were observed on other parameters such as fasting plasma insulin, glycated hemoglobin (HbA1c) or insulin sensitivity; of note, high heterogeneity between studies was observed for these outcomes. Higher doses of RS (≥ 26 g/d) compared to a lower dose (< 26 g/d) appeared more effective on FPG, as well as longer intervention duration (≥ 8 weeks vs. < 8 weeks). The effect was more pronounced in overweight subjects or those at risk of having diabetes, compared to healthy or diabetic subjects.

Aggregating data from 19 randomized controlled trials involving 1,014 individuals at risk of metabolic diseases (e.g., type 2 diabetics, prediabetics, overweight or dyslipidemic subjects) resulted in significant reductions in fasting plasma glucose [−4.28 (95% CI: −7.01, −1.55); *p* = 0.000; 14 studies], insulin [−1.95 (95% CI: −3.22, −0.68); *p* = 0.000; 12 studies] and HbA1c [−0.60 (95% CI: −0.95, −0.24); *p* = 0.000; 8 studies] due to RS interventions compared to digestible starches, other carbohydrates or other fibers. However, insulin resistance (HOMA-IR) was not significantly impacted (10 studies). Fasting plasma glucose or insulin resistance were significantly impacted in subjects with metabolic or renal diseases but not in at-risk subjects. Of note, using maize as a RS source appeared to not significantly affect FPG (61).

Gao et al. included 14 articles with a total of 515 subjects in their meta-analysis. Individuals presented obesity (6 studies) or had type-2 diabetes mellitus (T2DM) (6 studies) or without (2 studies) obesity. The studies included all forms of RS, and control typically consuming unfermented digestible carbohydrates. No significant effects of RS supplementation on either body mass index (BMI) or fasting plasma glucose were found after pooling 8 and 11 studies, respectively. However, a significant reduction was noted in patients with T2DM and obesity [−0.19 (95% CI: −0.29, −0.10); *p* < 0.0001; 5 studies]. Fasting plasma insulin was significantly lower in the RS supplementation group compared to the controls [−2.07 (95% CI: −3.25, −0.89); *p* < 0.0006; 8 studies], albeit with high heterogeneity (*I*^2^ = 84%). A more significant reduction was observed in the subgroup receiving 10 g/d of RS versus 30–40 g/d. Insulin sensitivity (HOMA-IR) was significantly reduced versus control only in the subgroup with T2DM [−0.71 (95% CI: −1.23, −0.20); *p* < 0.007; 4 studies], and that of T2DM with obesity [−0.91 (95% CI: −1.36, −0.45); *p* < 0.0001; 8 studies] (59).

In another meta-analysis, the investigators focused on 20 studies (670 participants) administering RS from high amylose maize starch (type 2) at doses ≥8 g per day (66). Participants included healthy individuals, as well as those overweight/obese, or with metabolic syndrome or type-2 diabetes mellitus. No significant effect of RS against placebo was reported on fasting plasma glucose, based on 15 studies. Subgroup analyses by health status did not reveal any significant changes. Similarly, HbA1c and HOMA-IR appeared unaffected by RS compared to placebo. However, body weight was significantly reduced compared to control [−1.19 (95% CI: −2.27, −0.12); *p* < 0.03; 6 studies], with subgroup analysis indicating this effect was heavily influenced by studies focusing on type-2 diabetics.

Finally, Wang et al. reported that RS administered at doses ranging from 10 to 45 g per day to 428 overweight or obese participants from 13 randomized controlled trials significantly reduced fasting plasma glucose [−0.26 (95% CI: −0.5, −0.02); *p* = 0.035; 12 trials], insulin [−0.72 (95% CI: −1.13 to −0.31); *p* = 0.001; 10 trials], and glycated hemoglobin [−0.43 (95% CI: −0.74, −0.13); *p* = 0.005; 4 trials] compared to control, in both diabetic and non-diabetic subjects. However, no significant changes were reported for HOMA-IR (68).

#### Resistant starch and lipoproteins response

A meta-analysis specifically examined the effects of RS on blood lipids through randomized controlled trials. This pooled analysis included 14 studies with a total of 820 participants, both healthy or others, who were administered RS (mostly of type 2) at a dose ranging from 10 to 66 g per day. The authors reported significant decreases serum total cholesterol [−7.33 mg/dL (95% CI: −12.15, −2.52 mg/dL); 19 trials] and Low-Density Lipoprotein Cholesterol (LDL-C) [−3.40 mg/dL (95% CI: −6.74, −0.07 mg/dL); 16 trials] compared to control. Serum triglycerides and High-Density Lipoprotein Cholesterol (HDL-C), however, remained unaffected. The authors noted that longer RS supplementation periods (>4 weeks) had a more significant impact on total cholesterol and LDL-C levels. A higher dose (>20 g/d) of RS also appeared to lower triglyceride levels (71).

Other meta-analyses, initially focusing on glycemic response, also reported findings on lipoproteins. One such meta-analysis reported that RS significantly reduced serum total cholesterol [−8.19 (95% CI: −15.38, −1.00); 13 trials] and LDL-C [−8.57 (95% CI: −13.48, −3.66); 10 trials] while HDL-cholesterol and triglycerides remained unaffected compared to control (61). Snelson and colleagues, however, did not observe a significant effect of RS on blood lipoproteins (66), while another meta-analysis reported a significant reduction LDL-C levels [−0.35 (95% CI: −0.61 to −0.09; *p* = 0.008; 6 trials)] (68).

#### Resistant starch and inflammatory response

A total of 3 papers reporting the impact of RS on inflammatory mediators were retrieved by the present review.

One pooled result from 16 trials, primarily using RS2 (13 out of the 16 studies). The RS dose ranged from 6 g/d to 27 g/d, with controls being digestible cornstarch, manioc or maltodextrin. Subjects included those with type-2 diabetes, end-stage renal disease, chronic kidney disease, or at risk of having diabetes, along with two trials involving healthy participants. Significant improvements were observed in total antioxidant capacity [2.64 (95% CI: 0.34, 4.94); *p* = 0.03; 3 trials], and blood malondialdehyde [−0.55 (95% CI: −0.94, −0.17); *p* = 0.01;6 trials] in the intervention groups vs. control. Regarding inflammatory biomarkers, a significant reduction in blood C-reactive protein was observed in individuals with T2DM [−0.35 (95% CI: −0.65, −0.05); *p* = 0.02; 3 trials], but not in other subjects. Similarly, interleukin-6 [−0.90 (95% CI: −1.36, −0.45); *p* < 0.01; 3 trials] and tumor necrosis factor alpha (TNF-α) [−0.55 (95% CI: −1.02, −0.09); *p* = 0.02; 4 trials] levels were significantly reduced compared to control. Others parameters were not significantly altered between intervention and control groups (63).

Haghighatdoost et al. performed a meta-analysis of 8 randomized controlled trials (RCTs) including a total of 308 individuals. Four studies included individuals with renal disease, 3 with diabetes, prediabetes, or diabetes risk factors, and one study focused on overweight or obese individuals. Included studies investigated RS2, more specifically high amylose maize RS (“Hi-maize® 260”). Control food was mostly waxy corn starch, manioc or regular wheat flour. No significant effect of RS2 on hs-CRP or IL-6 could be observed based on a meta-analysis of 7 and 4 studies, respectively. Finally, a small but significant decrease in TNF-α levels following RS consumption was noted compared to control, [−0.003 pg./mL (95% CI: −0.004, −0.001); *p* < 0.0001; 4 trials], though with significant heterogeneity (*I*^2^ = 98.0%). The authors noted that the effect of RS on CRP was significantly more pronounced when considering studies using an intervention dose above 20 g/d compared to less than 20 g/d. Similar observations were made for overweight compared to normal-weight individuals and for studies with an intervention period of 6 weeks or more, compared to a shorter period. Overall, the authors concluded that RS failed to significantly reduce inflammatory mediators (60).

According to another meta-analysis, only TNF-α was significantly reduced following RS administration compared to control [−2.02 (95% CI: −3.14, −0.90); 5 trials] (61).

Pooling results from a total of 13 randomized controlled trial involving 672 subjects overall, Vahdat et al. evidenced that RS administered to individuals at doses ranging from 10 to 45 g per day did reduce significantly inflammatory biomarkers such as IL-6 [−1.11 (95% CI: −1.72, −0.50); 7 trials] or TNF-α [−2.19 (95% CI: −3.49, −0.90); 8 trials], though not CRP, whatever the subjects’ conditions (healthy, healthy overweight, type-2 diabetics, hemodialytics) (67).

Finally, it was reported in a pooled analysis of 16 randomized controlled trials, including a total of 739 individuals, that RS intake decreased significantly TNF-α [−0.711 (95% CI: −1.227, −0.194); *p* = 0.007] and IL-6 [SMD: −0.609 (95% CI: −0.924, −0.294); *p* < 0.001]. A significant increase in total antioxidant capacity (TAC) was also reported in the RS intervention group versus control [2.543 (95% CI: 0.069: 5.017); *p* = 0.044)]. No significant changes on other parameters were noted, except for CRP levels that were significantly reduced, though only in the subgroup of diseased subjects (69).

## Discussion

The available evidence, as summarized in this narrative review, suggests that RS may have a positive impact on health parameters related to glycemic control in individuals with impaired metabolic control. However, consensus has not been firmly established for the general population or for other outcomes such as inflammatory response or variations in lipoproteins. Notably, the meta-analyses included in this review mostly considered RS as a whole, and did not allow comparisons of RS types. Nonetheless, Pugh et al., in their subgroup analyses, highlighted that significant effects on glycemic response were primarily observed with RS1 and RS2 but not with RS3. This observation can be attributed to the fact that RS1 and RS2 received more extensive research attention across the scientific literature, whereas the analysis of RS3 was based on only two studies (64). A limited number of clinical trials also focused on RS4. For instance, in a randomized controlled trial involving 38 healthy adults, Gourineni et al. reported that a RS4 nutritional bar significantly decreased postprandial glucose compared with a control bar (74). Similar conclusions were drawn in another study comparing a RS4 muffin with a control muffin in 28 healthy individuals (75), while mixed results were reported in another study (76). Additionally, increased short-term satiety was reported after consumption of a RS4 scone compared to a standard scone (77). Interestingly, Cai et al. highlighted that factors such as a high amylose content, less-gelatinized starch, the presence of retrograded starch, and the maintenance of a larger particle size were significant determinants of an attenuated glycemic and insulinemic response in healthy individuals (78).

Overall, the benefits of the different types of RS are likely exerted through different pathways (64), as demonstrated by the varying microbiota responses to different types of RS. For instance, after 12 weeks of consuming 12 g of RS4 daily, a significant decrease in bile acid in stools were reported compared to control (79). In an *in vitro* study, both RS2 and RS4 in rice sticks led to significantly higher counts of *Bifidobacterium* and *Lactobacillus* compared to the control food under pH-controlled batch culture conditions. While the bifidogenic effect of RS2 seemed stronger with RS2, *Lactobacillus* counts were maintained longer with RS4. Of note, selective suppression of *Clostridium* was shown with RS4, while RS2 seemed to target *Bacteroides*. Moreover, both RS2 and RS4 significantly increased production of total SCFA and butyric acid compared to the control, though the concentration was highest with RS2 after 24 h fermentation (80). Another study by Li et al. also investigated the human microbiota response to RS2 (Hi Maize^®^ 260), RS3 (Novelose^®^ 330) and RS4 (Fibersym^®^ RW) through *in vitro* culture and metaproteomic, and reported that responses seemed highly dependent on the individual microbiome characteristics. Nonetheless, a shift in microbiome response was highlighted following RS2 and RS3 culture. Both types had similar abilities to increase butyrate-producing bacteria, possibly because both products originated from high-amylose maize, according to the authors. RS3 significantly increased protein production of *Bifidobacteriaceae* and *Ruminococcaceae* (81). It is worth noting that this significant inter-individual variation in microbiota response to RS has already been reported (82). Among the different types of RS, RS5 has evolved and is still under investigation. Complexation of RS with lipids has the potential to present high resistance to enzymatic hydrolysis, and more stability than RS3 (83). RS5 has also potential as a functional ingredient as replacement of fat in white pan bread was shown to reduce bread energy value and delay retrogradation while maintaining acceptable bread characteristics (40). Accordingly, rice starch-lipid complex has been shown to improve body weight, dyslipidemia and SCFAs production in obese rats (84), and to dampen glycemic response in mice (85). Furthermore, the recent developments related to the classification of RS5, and the extension of possible complexes with other components than lipids raises the potential of RS5 has a health promoting ingredient. Owing to their V-type crystalline structure, most RS5 complexes presents high resistance to digestion, though to various extents depending on the non-starch component (14). Wheat starch complexation with gluten or with pea protein hydrolysates was reported to hinder α-amylase activity (86, 87). A complex of rice starch with xanthan gum and locust bean gum undergoing HMT was shown to significantly raise RS content (88). Research continues on the identification of starch complex components that would present the best characteristics to constitute a health-promoting ingredient.

Overall, the complexity of establishing whether RS confers health benefits arise from both the variability in inter-individual response and the different metabolic pathways affected by the different RS types. Furthermore, different modifications of starch structures during food processing or physical treatments also impact physiological response differently. From most of the studies presented in this review, none really correlated RS effects on health to the food process used.

Despite all these variables, the documented benefits of RS on health cannot be dismissed. The European Food Safety Authority (EFSA) established that “health claims related to the benefits of RS on postprandial glycemic response may be made when digestible starch in a given food has been replaced by RS so that the final content of RS is at least 14% of total starch” (89). While much of the evidence belong to RS2 from high amylose maize, the EFSA considered that all sources of RS would achieve a similar effect, as it would consist in replacing digestible CHO by indigestible CHO.

In conclusion, according to the current state of the literature, the relationship between RS and health appears to be multifaceted, with different RS types exerting distinct effects. Further research is needed to comprehensively characterize the specific properties and mechanisms of action of various RS forms.

## Author contributions

NTB: Visualization, Writing – review & editing. RD: Conceptualization, Investigation, Writing – original draft, Writing – review & editing, Data curation, Methodology. A-KI: Writing – review & editing, Methodology. PV: Writing – review & editing. LR: Writing – review & editing. PA: Writing – review & editing. MD-D: Conceptualization, Funding acquisition, Supervision, Visualization, Writing – original draft, Writing – review & editing.
